# Quality of main types of hunted red deer meat obtained in Spain compared to farmed venison from New Zealand

**DOI:** 10.1038/s41598-020-69071-2

**Published:** 2020-07-22

**Authors:** Martina Pérez Serrano, Aristide Maggiolino, Tomás Landete-Castillejos, Mirian Pateiro, Javier Pérez Barbería, Yolanda Fierro, Rubén Domínguez, Laureano Gallego, Andrés García, Pasquale De Palo, José Manuel Lorenzo

**Affiliations:** 1 Animal Science Techniques Applied to Wildlife Management Research Group, Instituto de Investigación en Recursos Cinegéticos, Universidad de Castilla-La Mancha, 02071 Albacete, Spain; 2 Sección de Recursos Cinegéticos y Ganaderos, Instituto de Desarrollo Regional, Universidad de Castilla-La Mancha, 02071 Albacete, Spain; 3 Departamento de Ciencia y Tecnología Agroforestal y Genética, Escuela Técnica Superior de Ingenieros Agrónomos y Montes, Universidad de Castilla-La Mancha, 02071 Albacete, Spain; 4 Department of Veterinary Medicine, University of Bari A. Moro, 70010 Bari, Italy; 5 Centro Tecnológico de la Carne de Galicia, 32900 Ourense, Spain; 6 Yolfi Properties S.L., 13180 Ciudad Real, Spain

**Keywords:** Ecology, Zoology

## Abstract

Deer venison is increasingly valued as a natural meat. This study examines the three main sources of venison: farmed venison from New Zealand (NZ), the world’s leading producer, and wild deer from Spain (SP), the second largest producer, which mainly supplies venison from traditional autumn–winter driven hunts (monteria), involving packs of dogs, and a smaller proportion culled through summer selective stalking. Meat from NZ contained more protein, lower shear force and lower *n*-6/*n*-3 ratio (*P* < 0.01). Spanish meat had a greater content of total, essential and non-essential amino acids (*P* < 0.01). Meat from deer culled in winter had lower intramuscular fat and saturated fatty acids (FA) but higher polyunsaturated FA (*P* < 0.01) and pH (*P* < 0.001) than meat from summer stalked deer. Therefore, venison presents differences by country of origin for composition, FA and texture that are likely to affect its health characteristics. Anomalies observed in meat between the winter driven hunt and non-stressful summer stalking may be attributed to the level of death stress in the case of variables such as pH. However, the effect on fat and mineral composition seems to be seasonal, depending respectively on wild diet or cyclic osteoporosis in males.

## Introduction

During the last decade, consumption of red deer meat has increased^1,2^. Of the total 12 million population of red deer (wild and farmed), 440,000 deer are slaughtered for meat each year, mostly by hunting^2^. New Zealand (NZ), the world leader in farmed deer meat, has an estimated one million farmed deer (compared to four million inhabitants)^2^. Spain (SP), the second largest exporter of venison, produces 11,250 tonnes of venison almost entirely sourced from trophy hunting^2^. Although most of SP meat is produced in stressful driven hunts (monteria) during autumn and winter, selective stalking (low-level stress death) in SP and other countries during summer yields a second source of meat in through culling poor trophy animals or to reduce population density. Thus, inevitably, the comparison between the most common SP marketed meats shows a mixed effect of hunting type with seasonality: the driven hunts during winter vs. summer when wild animals have a rather different amount of food available and stalking results in sudden death rather than the extreme stress of being chased by dogs. A literature review focusing on quality of venison shows that the animals studied (farm-raised or wild deer) were slaughtered/hunted during one particular season of the year, being either the autumn or the winter time^3^. There is very little available information about meat quality from deer hunted in summer. Cifuni et al.^4^ assessed the effects of different methods of hunting on meat quality from fallow deer and wild boar. Cockram et al.^5^ examined several types of stalking vs. shooting of farmed red deer, but no information on traditional driven hunts, which involve packs of hounds. Thus, no studies so far have assessed the differences in meat quality between red deer slaughtered in driven hunts and stalking.

Farmed red deer meat from NZ has been widely studied and analysed^6–9^, as has meat quality of wild deer in SP sourced from driven hunts in autumn or winter^1,2,10^, but not from selective shooting in summer. So far, no study has made a comparison of the most common types of deer meat available in the market (again, studying the impact of stressful winter driven hunts and summer stalking in SP vs. farmed deer from NZ). Thus, the aim of this study was to assess for the first time the combined impact of country of origin (SP vs. NZ) and slaughter type/season (stressful driven hunts in winter vs. the sudden death of stalking in summer, sampling both in the same game estate to reduce confounding factors) on the quality and nutritional value of red deer meat.

## Results

### Meat quality traits

The country of origin did not influence pH, redness, intramuscular fat (IMF) and cholesterol contents or cooking losses (Table 1). However, meat from SP-wild deer presented higher values of lightness (*P* < 0.001), yellowness (*P* < 0.001), chroma (*P* < 0.05) and hue angle (*P* < 0.001) than meat from NZ-farmed deer. In addition, SP-wild meat had a higher content of moisture (*P* < 0.01) but lower levels of protein (*P* < 0.001) and ashes (*P* < 0.01) than NZ-farmed meat. However, meat from NZ farms had 58.7% lower shear force than meat from SP wild game (*P* < 0.001).

**Table 1:** Effects of country of origin (Spain vs. New Zealand) and hunting type most commonly used in Spain (stressful-winter vs. stalking summer) on quality traits and chemical composition of red deer meat.

Effect	Country of origin (OR)				Hunting type (HT)			
Item	Spain	New Zealand	SEM^a^	*P *value^b^	Stressful-winter	Stalking-summer	SEM^c^	*P *value^b^
pH	5.67	5.68	0.027	ns	5.78	5.55	0.037	***
**Colour traits**								
Lightness	35.3	29.1	0.82	***	34.8	35.7	0.84	ns
Redness	15.4	14.6	0.42	ns	14.1	16.6	0.68	0.07
Yellowness	11.8	8.9	0.45	***	10.9	12.7	0.53	0.08
Chroma	19.5	17.1	0.54	*	17.9	21.0	0.78	0.05
Hue angle	0.66	0.55	0.017	***	0.66	0.65	0.019	ns
**Chemical composition**								
Moisture (%)	75.2	73.5	0.28	**	76.5	73.8	0.39	***
Protein (%)	22.7	24.1	0.20	***	21.9	23.5	0.26	***
Fat (%)	0.51	0.75	0.087	ns	0.11	0.90	0.137	***
Ash (%)	1.18	1.33	0.026	**	1.07	1.28	0.035	***
Cholesterol (mg/100 g)	41.1	41.8	1.59	ns	40.5	41.6	2.18	ns
Cooking losses (%)	24.0	23.9	0.77	ns	20.5	27.4	1.19	***
Shear force (N/cm^2^)	42.9	25.2	2.39	***	46.5	39.3	2.34	ns

^a^*n* = 14 and 10 for Spain and New Zealand, respectively.

^b^*ns* not significant (*P* > 0.10); *P < 0.05; **P < 0.01; ***P < 0.001.

^c^*n* = 6 and 8 for stressful-winter hunted and stalking-summer, respectively.

In addition to a lower pH (*P* < 0.001), meat from summer stalking deer tended to present higher values for redness (*P* = 0.07), yellowness (*P* = 0.08) and chroma (*P* = 0.05) than meat from winter chase deer. However, no differences were detected for the dark, firm and dry (DFD) incidence (pH ≥ 6.0 for 0% of carcasses from both treatments), with pH ranging from 5.46 to 5.80. Moreover, meat from deer sourced from summer stalking presented higher cooking losses than meat from those slaughtered during winter driven hunts (*P* < 0.001) despite having higher contents of IMF. No differences between hunting types were observed for cholesterol content or shear force.

### FA methyl ester content

The country of origin did not influence the saturated (SFA) and polyunsaturated (PUFA) fatty acids (FA), PUFA/SFA ratio, nutritional value (NV), hypocholesterolemic/hypercholesterolemic ratio (h/H) and index of atherogenicity (IA; Table 2). However, meat from NZ farms tended to have a higher content of monounsaturated FA (MUFA) than meat from SP wild estate (*P* = 0.06). In addition, NZ farmed meat had higher (*P* < 0.001) contents of *n*-3 FA and long chain *n*-3 PUFA but less *n*-6 FA content (*P* < 0.05), *n*-6/*n*-3 (*P* < 0.01) and index of thrombogenicity (IT; *P* = 0.06) than SP wild meat. The higher content of MUFA for NZ farm meat was due to its higher content of vaccenic (C18:1*n*-7) and trans-vaccenic (TVA; C18:1C11t) acids (*P* < 0.001).

**Table 2:** Effects of country of origin (Spain vs. New Zealand) and hunting type most commonly used in Spain (stressful-winter vs. stalking summer) on fatty acid profile of red deer meat (g/100 g of total fatty acids).

Effect	Country of origin (OR)				Hunting type (HT)			
Item	Spain	New Zealand	SEM^a^	*P *value^b^	Stressful-winter	Stalking-summer	SEM^c^	*P *value^b^
C14:0	3.61	3.96	0.29	ns	2.05	5.16	0.50	***
C14:1*n*-5	1.04	1.44	0.17	ns	0.08	2.00	0.27	***
C15:0	0.37	0.53	0.023	***	0.30	0.44	0.023	***
C16:0	22.1	23.7	1.36	ns	13.9	30.2	2.35	***
C16:1*n*-7	6.45	7.7	0.70	ns	2.4	10.5	0.18	***
C17:0	0.53	0.63	0.021	**	0.60	0.45	0.027	***
C18:0	14.0	12.3	0.63	ns	17.6	10.4	1.05	***
C18:1*n*-7	2.00	3.58	0.20	***	1.40	2.61	0.18	***
C18:1*n*-9	14.1	14.9	0.31	ns	14.3	13.8	0.52	ns
C18:1C11t	0.49	1.27	0.096	***	0.58	0.40	0.031	***
C18:2*n*-6	15.5	9.9	1.15	*	21.3	9.6	1.81	***
C18:3*n*-3	3.84	5.35	0.28	**	4.21	3.46	0.31	ns
C20:3*n*-6	0.92	0.46	0.083	*	1.31	0.52	0.123	***
C20:4*n*-6	6.75	3.8	0.63	0.054	10.1	3.4	0.96	***
C20:5*n*-3	1.51	2.93	0.19	***	1.29	1.73	0.19	ns
C22:5*n*-3	2.69	2.93	0.13	ns	3.09	2.29	0.18	*
C22:5*n*-6	1.45	0.98	0.13	ns	2.17	0.72	0.21	***
C22:6*n*-3	0.77	1.35	0.11	**	1.24	0.30	0.14	***
SFA^d^	40.6	41.2	1.08	ns	34.4	46.7	1.84	***
MUFA^e^	24.0	28.8	1.06	0.06	18.7	29.3	1.68	***
PUFA^f^	33.4	27.7	2.05	ns	44.7	22.0	3.45	***
PUFA/SFA	0.90	0.67	0.075	ns	1.31	0.48	0.126	***
∑*n*-6	24.6	15.1	1.95	*	34.9	14.2	3.05	***
∑*n*-3	8.8	12.6	0.57	***	9.8	7.8	0.65	ns
Long chain *n*-3 PUFA^g^	4.97	7.21	0.356	***	5.62	4.31	0.363	0.07
∑*n*-6/∑*n*-3	2.78	1.22	0.229	**	3.71	1.85	0.303	***
Nutritional value^h^	1.00	1.12	0.091	ns	0.46	1.53	0.157	***
h/H^i^	2.23	1.38	0.236	ns	3.50	0.96	0.391	***
IA^j^	0.68	0.70	0.057	ns	0.35	1.00	0.097	***
IT^k^	0.82	0.66	0.049	0.06	0.60	1.03	0.077	***

^a^*n* = 14 and 10 for Spain and New Zealand, respectively.

^b^*ns* not significant (P > 0.10); *P < 0.05; **P < 0.01; ***P < 0.001.

^c^n = 6 and 8 for stressful-winter hunted and stalking-summer, respectively.

^d^Saturated fatty acids = C14:0 + C15:0 + C16:0 + C17:0 + C18:0.

^e^Monounsaturated fatty acids = C14:1*n-5* + C16:1*n-7* + C18:1*n*-*7* + C18:1*n*-*9* + 18:1C*11t.*

^f^Polyunsaturated fatty acids = C18:2*n*-*6* + C18:3*n*-*3* + C18:3*n*-*6* + C20:4*n*-6 + C20:5*n*-3 + C22:5*n*-3 + C22:5*n*-6 + C22:6*n*-3.

^g^C20:5*n-3* + C22:5*n-3* + C22:6*n-3.*

^h^Σ(C14:0 + C16:0)/Σ(C18:1*n*‒9 + C18:2*n*‒6).

^i^Hypocholesterolemic/hypercholesterolemic ratio = [Σ(C18:1*n*‒7 + C18:1*n*‒9 + C18:2*n*‒6 + C18:3*n*‒3 + C20:3*n*‒6 + C20:4*n*‒6)/Σ(C14:0 + C16:0)].

^j^Index of atherogenicity = [(4*C14:0) + C16:0]/[(∑MUFA) + (∑PUFA)].

^k^Index of thrombogenicity = [C14:0 + C16:0 + C18:0]/[(0.5*∑MUFA) + (0.5*n‒6) + (3*n‒3) + (*n*‒3/*n*‒6)].

The FA profile of SP wild meat samples varied with season/hunting type. In fact, the main FA in red deer meat from winter driven hunts were the PUFA (around 44.7 g/100 g of total FA), followed by the SFA (around 34.4 g/100 g of total FA) and the MUFA (around 18.7 g/100 g of total FA). However, the main FA of deer meat from summer stalking were the SFA (46.7 g/100 g of total FA) followed by the MUFA (29.3 g/100 g of total FA) and the PUFA (22.0 g/100 g of total FA) (*P* < 0.001). No differences were detected between hunting types for the *n*-3 FA content of meat. However, meat obtained in winter driven hunts tended (*P* = 0.07) to have higher content of long chain *n*-3 PUFA than meat obtained in summer stalking. Consequently, the fat of deer slaughtered during winter driven hunts presented higher PUFA/SFA ratio, *n*-6 FA content and *n*-6/*n*-3 ratio than summer stalking fat (*P* < 0.001).

Regarding the SFA, the dominant FA were myristic (C14:0), palmitic (C16:0) and stearic (C18:0). Meat from summer stalking had the higher contents of C14:0 and C16:0, while meat from winter driven hunts had higher contents of C18:0 (*P* < 0.001). With regard to MUFA, oleic acid (C18:1*n*-9) was the dominant FA (14.3 and 13.8 g/100 g of total FA as average for winter chase and summer stalking, respectively), but no differences were observed between hunting/season types. On the other hand, fat from winter driven hunts had more TVA, an important precursor of conjugated linoleic acid, than fat from summer stalking (*P* < 0.001). Among PUFA, the linoleic acid (C18:2*n*-6) was the main FA, followed by arachidonic acid (C20:4*n*-6) presenting both FA higher values in fat from winter driven hunt meat (*P* < 0.001). Finally, α-linoleic acid (C18:3*n*-3) and docosapentaenoic acid (C20:5*n*-3) showed no difference between hunting/season types.

### Amino acid (AA) profile

Among the essential AA fraction, the dominant AA was lysine, followed by arginine and leucine, together accounting for approximately 50% of total essential AA, whereas methionine presented the lowest values (around 2.4% of total essential AA; Table 3). Regarding the non-essential AA fraction, glutamic acid, aspartic acid and alanine were the main AA observed, representing together around 71% of the total non-essential AA, whereas glycine, serine and proline showed the lowest contents (around 29% of total non-essential AA).

**Table 3:** Effects of country of origin (Spain vs. New Zealand) and hunting type most commonly used in Spain (stressful-winter vs. stalking summer) on amino acids (AA) profile of red deer meat (expressed as mg/100 g of sample).

Effect	Country of origin (OR)				Hunting type (HT)			
Item	Spain	New Zealand	SEM^a^	*P* value^b^	Stressful-winter	Stalking-summer	SEM^c^	*P* value^b^
**Essential AA**								
Histidine	883	427	54.9	***	773	993	34.8	***
Threonine	1,081	1,151	16.9	*	1,077	1,084	20.4	ns
Valine	1,278	1,159	29.1	*	1,184	1,371	39.2	*
Methionine	295	295	6.4	ns	278	312	8.9	0.05
Lysine	4,315	2,081	53.8	ns	2,023	2,292	74.4	0.07
Isoleucine	1,216	1,025	29.2	***	1,170	1,261	31.3	ns
Leucine	2,043	1,778	45.1	***	1,992	2,094	51.8	ns
Phenylalanine	1,086	936	20.9	***	1,048	1,123	21.0	0.08
Tyrosine	834	602	25.5	***	839	828	15.2	ns
Arginine	2,072	1,833	40.3	**	2,179	1,964	51.9	*
**Non-essential AA**								
Aspartic acid	2,084	1,414	79.0	***	2,061	2,107	57.2	ns
Serine	883	1,015	24.0	**	800	966	30.7	**
Glutamic acid	3,635	3,324	79.9	0.07	3,746	3,523	101.6	ns
Glycine	987	886	19.1	**	978	996	22.9	ns
Alanine	1,299	1,213	28.4	ns	1,375	1,223	39.6	0.05
Proline	906	810	19.0	*	954	857	25.7	0.06
Total AA	22,734	19,951	480.6	**	22,476	22,992	537.3	ns
Essential AA	12,941	11,287	276.6	***	12,562	13,320	304.1	ns
Non-essential AA	9,793	8,664	211.6	**	9,914	9,672	250.4	ns
Essential/non-essential AA	1.33	1.30	0.011	ns	1.27	1.38	0.017	***

^a^*n* = 14 and 10 for Spain and New Zealand, respectively.

^b^*ns* not significant (P > 0.10); *P < 0.05; **P < 0.01; ***P < 0.001.

^c^n = 6 and 8 for stressful-winter hunted and stalking-summer, respectively.

Meat from SP wild deer had higher (*P* < 0.01) contents of total, essential and non-essential AA than meat from NZ farmed deer. No differences were observed between countries of origin in relation to essential/non-essential AA ratio. However, meat from summer stalking tended (*P* = 0.07) to have higher content of lysine and presented a higher (*P* < 0.001) essential/non-essential AA ratio than meat from winter driven hunts. No differences for total, essential and non-essential AA were observed between hunting types/season for SP meat samples.

### Mineral content

The main mineral identified was potassium (K) (ranging from 2.90 to 3.65 g/kg), followed by phosphorus (P), ranging from 1.93 to 2.34 g/kg (Table 4). Among trace-minerals, zinc (Zn), ranging from 20.1 to 56.4 mg/kg, and iron (Fe), ranging from 27.0 to 34.1 mg/kg, were the most abundant. No differences were detected between countries of origin with regard to calcium (Ca), sodium (Na) and P contents. However, meat sourced from NZ farms contained more (*P* < 0.05) K, magnesium (Mg), Fe, manganese (Mn) and copper (Cu) and less Zn than meat from SP wild deer. The mineral profile of meat varied with the hunting type/season: thus, meat from winter driven hunts had higher contents of Ca, Na, Fe and Zn and lower contents of Mg and P compared to meat sourced from summer stalking (*P* < 0.05).

**Table 4:** Effects of country of origin (Spain vs. New Zealand) and hunting type most commonly used in Spain (stressful-winter vs. stalking summer) on mineral content of red deer meat.

Effect	Country of origin (OR)				Hunting type (HT)			
Item	Spain	New Zealand	SEM^a^	*P *value^b^	Stressful-winter	Stalking-summer	SEM^c^	*P *value^b^
**Macrominerals, g/kg**								
Calcium	0.038	0.038	0.0011	ns	0.044	0.032	0.0018	***
Potassium	2.97	3.65	0.085	***	3.03	2.90	0.062	ns
Magnesium	0.22	0.37	0.017	***	0.20	0.24	0.0056	***
Sodium	1.25	1.20	0.043	ns	1.43	1.06	0.063	***
Phosphorus	2.14	2.29	0.040	ns	1.93	2.34	0.061	***
**Trace-minerals, mg/kg**								
Iron	29.5	34.1	1.06	*	31.9	27.0	1.18	*
Manganese	0.14	0.17	0.005	**	0.15	0.13	0.005	ns
Zinc	39.2	20.1	3.28	**	56.4	22.0	4.80	***
Copper	1.45	2.04	0.082	***	1.34	1.56	0.074	ns

^a^*n* = 14 and 10 for Spain and New Zealand, respectively.

^b^*ns *not significant (*P* > 0.10); *P < 0.05; **P < 0.01; ***P < 0.001.

^c^*n* = 6 and 8 for stressful-winter hunted and stalking-summer, respectively.

## Discussion

A number of studies have assessed meat sourced from wild deer originating from various countries (i.e.^11^^,^^12^ from Poland or^13^ from South Africa) or from farmed deer (i.e.^14^ from Czech Republic^11^^,^^15^, from Poland^16^, from Italy and^17^from SP). Studies have also examined the main marketed deer meats: meat from wild SP deer^1,2,10^ and farmed NZ ones^6–8,18^. Despite the number of studies cited below which assess meat quality from different countries or different types of breeding/killing (wild or farmed, stressful or sudden death), they have been undertaken by different research teams, using different scientific equipment and protocols. None of these studies have assessed meat samples from different countries with the same scientific equipment, reagents, and staff. It is therefore likely that some of the anomalies found through comparison of the available literature may actually be caused by methodological rather than regional differences per se.

The quality of wild game meat depends on the hunting method^19^ and on the hunting season^3^. Wild deer shot with a projectile (e.g. a bullet) are not exsanguinated immediately after their death and often several hours elapse between death and dressing. Consequently, carcasses are often processed once *rigor mortis* has set in, which affects meat characteristics. In principle, a low stress death by stalking should result in a better quality of meat and, in fact, meat-processing companies pay better prices for meat from stalked animals than for stress hunted meat, as evidenced by game estate owners and personal interviews. Studies have assessed several types of hunted and farmed venison, but none included stressful pursuit by dogs. Our results show for the first time that the mixed effects of hunting type-season resulted in some differences in meat quality (pH, cooking losses), but surprisingly not in tenderness (shear force).

Recently, Stanisz et al.^3^ have not observed differences for body weight with hunting season of hunt-harvested does. However, the authors observed a higher technological quality for venison obtained in winter showing compared to that harvested in summer. In fact, it was observed lower purge loss in vacuum, drip loss, free water, free water share in total water, and water loss during roasting. In addition, venison obtained in winter showed a greater brightness and a reduced redness comparing to venison obtained in summer. Colour traits and water retention capacity determine the meat shelf life and its suitability for storage in vacuum packaging.

Values obtained in the current study for pH of meat were similar to those observed previously for meat from NZ farmed deer^7,9^ and for SP stressed, hunted red deer^2,10^. However, no data has been found to compare the average values of pH obtained in SP from stalked deer. Our results show that pH values were similar for wild deer in SP (pooled values) and farmed deer in NZ, but meat from stalked deer had the lowest values. This is not surprising as transporting deer to the abattoir also involves stress, and furthermore, recently, Gentsch et al.^20^ have observed that cortisol levels for stalked deer were much lower than those for deer hunted with dogs in driven hunts (21.8 vs. 66.1 nmol/L, respectively). With regard to the effect of seasons, meat hunted in winter had a higher pH than meat hunted in summer. However, recently, Stanisz et al.^3^ observed that the pH of the muscles *Longissimus lumborum* measured 24 h post-mortem was 0.22 units higher in the summer season, compared to the winter season. This is consistent with current results regarding colour and literature reports that pre-harvest stress affects the degree of bleed, leading to an increase in the level of oxymyoglobin^21^ and confirming the influence of hunting type on meat colour^4^. According to results obtained by Stanisz et al.^3^, venison sourced from does shot in summer was redder and had a greater chroma compared to venison obtained in winter. In our study, values obtained for colour traits were similar to those obtained for NZ farmed^6,9^ and for SP stress hunted^10^ deer meat.

The IMF content was similar to the values previously reported for NZ farmed deer^8,9^ and for autumn–winter hunted deer from the same region of SP^1,10^. However, the most interesting information came from slight differences in IMF between seasons/type of hunting in wild SP: meat from summer stalking had an average IMF content of 0.90%, a value significantly higher than that for winter chased meat (0.11%). This may be only a seasonal difference (unlikely to be attributable to stress at death) because of increased grazing available during spring and summer for the deer. Thus, it is well established that body condition of deer improves, mainly in gaining fat and body weight, during spring and summer. In contrast, loss condition, involving loss of body fat, is higher in autumn and winter^22^. In addition, deer lose weight in autumn because feed intake decreases considerably during the rut^23^. Confirming this hypothesis, Serrano et al.^2^ have found higher contents of IMF and cholesterol in the loin of deer hunted in driven hunts in autumn compared to the loin from deer hunted in driven hunts in winter. The cooking losses of meat were similar to values reported previously for NZ farmed deer^7^ and for stressed deer hunted in driven hunts in SP^10^, although there was an effect of stress/season with values higher for stalking-summer. The effect of season on cooking losses has been previously described^3^. However, the cause of the differences observed in current study is likely to be due to the level of stress at slaughter, corroborated by Cifuni et al.^4^, who found that meat from culled (selective hunting) deer produced a greater degree of water loss during cooking than meat from deer slaughtered in driven hunts.

In general, values reported for shear force present a high variability^2,12,13^. Values in the current trial were higher than those reported previously for deer meat^1,8–10^. Differences among authors might be due to a range of interrelated factors, including pH, amount of connective tissue, IMF content, proteolytic enzyme activity and age of the animal^13^. Differences observed for the shear force between countries of origin (58.7% higher for meat from SP than for NZ meat) are not caused by stress at death: no differences were observed regarding the shear force of SP meat by hunting type/season. In fact, Stanisz et al.^3^ concluded that a greater impact of season could be evidenced using biting measures (Volodkevich Bite Jaws, test speed 2 mm/s; strain 100%; force 5 g at 24 h and 14 days post-mortem) compared to Warner–Bratzler measures. However, biting measures were not included in the current study. Further studies may be needed to conclude whether there is an effect of season compensating for the apparent effect of stress that yields non-significant results.

In general, the country of origin did not influence the total content of SFA or PUFA and a trend was only detected in the increase of the MUFA content of NZ meat. However, meat from NZ farms had higher content of *n*-3 FA and long chain *n*-3 PUFA, less content of *n*-6 FA and, in consequence, a lower *n*-6/*n*-3 ratio than SP wild meat. Values obtained for the *n*-6/*n*-3 ratio (ranging from 1.22 to 3.71) correspond with those reported by other authors for deer meat^8,14^. In any case, the average *n*-6/*n*-3 ratio for meat from both countries of origin was lower than 4, as recommended by WHO/FAO^24^.

The FA profile differed for meat samples obtained by different hunting types/season. The differences observed in the current study between hunting types for the FA profile are likely to be caused by the effects of the diet FA profiles seasonal changes on ruminant products^25^. Thus, the main FA in winter driven hunt meat were PUFA, followed by SFA and MUFA. In summer stalked deer and farmed venison, the main FA were SFA followed by MUFA and PUFA. No differences were observed for the contents of *n*-3 FA. Consequently, the fat of animals sourced from driven hunts in winter showed a higher PUFA/SFA ratio, *n*-6 FA content and *n*-6/*n*-3 ratio than the fat of summer stalked deer.

In general, the AA profile obtained in this study corresponds with that reported for meat from red deer, as previously indicated by Lorenzo et al.^1^. Interesting effects of country of origin and hunting type on AA profile of deer meat were observed in the current study. The SP wild meat had higher contents of total, essential and non-essential AA than NZ farm meat. Moreover, meat collected from summer-stalked deer presented a higher ratio for the essential/non-essential AA than meat collected from deer slaughtered in winter driven hunts. However, authors have not found previous studies comparing the AA profile of meat from SP and NZ deer slaughtered by different methods to compare with current results. It is postulated, therefore, that AA differences are attributable to seasonal effect, not the level of stress at death.

Because mineral composition presented in deer meat is closely related to the natural environment as they graze and browse^26^, differences found in mineral content in our study seem to depend on season and diet composition rather than on level of stress at death. Therefore, the differences in fodder available in spring and summer as compared to winter, as well as the growth of leaves in deciduous trees and shrubs, may explain some of the differences in the mineral profile observed between meats sourced from winter driven hunts and summer stalking analysed in the current study. Actually, Estévez et al.^27^ found seasonal differences in the mineral content of plants consumed by deer in SP. It is highly unlikely that mineral differences between both meats are due to the level of stress at death: the only difference that could be expected (Na content) due to increased sweating resulting from the chase and stress, was actually the reverse of what was expected (greater in animals killed in winter driven hunts). These results suggest that the difference was due to a lower level of Na in plants in summer.

One of the most significant effects in the seasonal differences found in the current study may not be related to mineral content of the diet, but to another very interesting and unique physiological characteristic of male deer (the sex examined in this study): cyclic physiologic osteoporosis. This effect is caused by rapid growth of the antlers (more than 1 cm/day), causing a depletion of mineral stores in certain bones in order to transfer the material to antlers^28^. Because minerals are blood borne, it is not surprising that they may also affect the mineral composition of muscles. This could explain why meat from osteoporotic deer (summer) had less than half the content of Zn, which forms part of alkaline phosphatase, the enzyme needed to deposit Ca in bone tissue^29^. For the same reason, it may explain the differences found for P and Ca contents (despite Ca is more stable in blood), and even for Mg (which can substitute Ca in the hydroxyapatite forming the antlers and bones).

## Methods

### Experimental design

Meat samples from SP were obtained from 14 red deer controlled for age (adults from 3 to 7 years of age), and homogeneous in size and sex (males). Age was determined for each carcass by three independent trained persons (wildlife veterinarians not connected with the authors of the paper) who followed guidelines reported by Brown and Chapman^32^ using eruption evaluation, wear patterns and wear score of mandibular molars. In 11 of the carcasses, the age estimates by the three evaluators were identical. Estimates for the remaining carcasses were slightly different and the age assigned was the arithmetic mean between the values supplied by the assessors.

From these, six deer were hunted in January (winter in SP) following the most common type of driven hunt in SP, involving a chase by dogs (stressful death), while eight deer were subjected to sudden-low stress death by stalking in mid-June (beginning of summer in SP), when selective shooting is permitted to cull poor trophies and reduce population density. All SP individuals were hunted on the same game estate (Ciudad Real, south central Spain) to minimise variability caused by local or regional effects. Animals were exsanguinated, eviscerated and decapitated at the atlanto-occipital junction in the countryside. Whereas more than 5 h elapsed between death and processing of carcasses in the winter driven hunts, less than 1 h elapsed between death and carcasses processing in summer-stalking.

Samples of *Longissimus thoracis et lumborum* (LTL) from T8 to L6 muscle were collected from each carcass, vacuum packed and transported to the laboratory (Centro Tecnolóxico da Carne, Ourense, Spain) in refrigerated conditions. Meat samples from SP were compared with LTL separated from commercially available loins (Australian Gourmet S.L., Barcelona, Spain) from 10 male deer from NZ-farm (slaughtered in April, autumn in the southern hemisphere). All meat samples (SP and NZ) were stored at 2–3 °C until analysis.

### Meat quality traits

Each LTL sample was divided into six steaks. The first three steaks were used to determine pH, colour traits and chemical composition. The fourth and fifth steaks were used to determine the cooking losses and the shear force, respectively, whereas the sixth steak was used for the analysis of cholesterol, FA, AA and minerals.

Analytical methods used for pH, incidence of DFD meats (pH ≥ 6) and colour are described in Maggiolino et al.^10^. External fat was subsequently removed from each sample and meat was minced and mixed to produce a homogeneous mixture. Chemical composition, cooking loss and shear force were then measured according to Maggiolino et al.^10^. The determination of total cholesterol, FA methyl esters, AA profile and mineral content of LTL was conducted as described by Lorenzo et al.^1^. The FA data was used to calculate the total content of SFA, MUFA and PUFA, the PUFA/SFA ratio, the total content of *n*-6, *n*-3 and long chain *n*-3 PUFA and the *n*-6/*n*-3 ratio. Additionally, lipid quality indices (nutritional value; hypocholesterolemic/hypercholesterolemic ratio; index of atherogenicity and index of thrombogenicity) were calculated as indicated by Lorenzo et al.^1^.

### Statistical methods

Two general linear model (GLM) tests were performed. The first GLM analysis enabled the study of effects of country of origin and wild/farmed deer (SP vs. NZ) (fixed effect) on meat quality characteristics and nutritional value traits (dependent variables). The second GLM test examined the effects of hunting type/season (stressful hunted in winter vs. non-stressful death in summer stalking) (fixed effect) on meat quality and nutritional value traits (dependent variables) from SP deer meat. Differences were considered significant at *P* < 0.05. In all cases, the experimental unit was the individual animal (*n* = 14 and 10 for SP and NZ, respectively and *n* = 6 and 8 for stressful hunted in winter and stalking in summer, respectively). The mean value obtained for each trait from 6 steaks collected from each 14 SP deer and from 6 steaks collected from each 10 NZ deer was calculated. Values are given as means and standard error of mean. All analyses were carried out with SPSS version 22.0 (2013) (SPSS Inc., NY, USA).

### Ethics statement

The animals subject to this study were handled according to ethical standards and existing legal regulations. The slaughter of the hunted animals was regulated by the Regional Hunting Law of Castilla la Mancha [^30^ modified by^31^].

## Conclusion

It can be concluded that meat from NZ farms had a higher content of protein and lower shear force and *n*-6/*n*-3 ratio (although this ratio was below than 4 for both countries of origin) than wild meat from SP. However, SP wild meat had a higher content of total and essential AA. With regard to the type of hunt, differing in season and stress through hunting, meat from deer slaughtered in winter at driven hunts had a lower content of IMF, which may be considered healthier as result of the lower content of SFA and the greater content of PUFA. Comparisons between deer meat sourced from driven hunts in winter and summer meat obtained by stalking can be explained (in the case of variables such as pH) as the result of the type of death, but most differences appear to be caused either from optimum body condition in summer, or from the mineral mobilization from the skeleton to grow antlers in males. Further studies are required to explain the origin of trends in seasonal variability and as well as those caused directly by stress at slaughter. However, current results show for the first time a picture of characteristics of most common marketed deer meat from both main producer countries.
